# Spatial thinking as the missing piece in mathematics curricula

**DOI:** 10.1038/s41539-022-00128-9

**Published:** 2022-06-02

**Authors:** Katie A. Gilligan-Lee, Zachary C. K. Hawes, Kelly S. Mix

**Affiliations:** 1 University of Surrey, Guilford, UK; 2 Centre for Educational Neuroscience, University of London, London, UK; 3 University of Toronto, Toronto, ON Canada; 4 University of Maryland, College Park, MD USA

**Keywords:** Human behaviour, Education

## Abstract

It is well established that spatial thinking is central to discovery, learning, and communication in mathematics, as indicated by convincing evidence that those with strong spatial skills also demonstrate advantages for Science, Technology, Engineering and Mathematics (STEM) performance. Yet, spatial thinking—the ability recall, generate, manipulate, and reason about spatial relations—is often absent from modern mathematics curricula. In this commentary, we outline evidence from our recent meta-analysis, demonstrating a causal role of spatial thinking on mathematics. We subsequently discuss the implications of educational policy decisions made across different countries, regarding the prioritization of spatial reasoning in the classroom. Given the increasing global demand for highly qualified STEM graduates, and evidence that spatial skills promote improvements in STEM outcomes, we argue that it is remiss to continue to ignore spatial skill development as a component of educational policy.

To engage in mathematics is to engage in spatial thinking. The Pythagorean Theorem, the Cartesian coordinate system, triangular numbers, the real number line, and Cavalieri’s principle offer but a few examples of the centrality of space in mathematics. Indeed, spatial thinking lies at the heart of what it means to discover, learn, and communicate mathematics, as indicated by convincing evidence that those with strong spatial skills also demonstrate advantages for Science, Technology, Engineering and Mathematics (STEM) performance^1^. Acknowledging the spatial-STEM association is particularly pertinent given the increasing importance of STEM industries in economic growth internationally, and the fact that many countries have identified shortages in qualified STEM graduates. Thus, improving STEM outcomes has become an economic priority^2,3^. Yet, spatial thinking—the ability recall, generate, manipulate, and reason about spatial relations—sits curiously in the shadows of present-day mathematics curricula. In some parts of the world, there are even initiatives to remove aspects of spatial thinking from the mathematics classroom. Here we argue this is a bad idea; one that ignores the historical roots of mathematics and the present-day empirical science on the contribution of spatial thinking in mathematics learning. In support of this claim, we provide new evidence demonstrating a causal role of spatial thinking on mathematics and discuss the implications of differing educational policy decisions made across the world, regarding the prioritization, and indeed deprioritization, of spatial reasoning in the classroom. Lastly, we consider how spatializing the mathematics curriculum may contribute to more accessible and equitable approaches to mathematics and STEM education.

Historically, research on the causal effect of spatial cognition on math has been driven by two key observations; (1) spatial thinking is a cognitive skill that is particularly malleable through training; and (2) there is extensive correlational evidence linking spatial skill to mathematics performance. In their 2013 systematic meta-analysis, Uttal and colleagues highlighted the immense potential of spatial constructs as cognitive intervention targets. Based on an analysis of 206 training studies across a 25-year period they reported that spatial interventions led to an average effect size (Hedges’s *g*) of 0.47 (half a standard deviation) for training groups relative to controls^4^. In an accompanying review article, they argued that “including spatial thinking in STEM curricula could substantially increase the number of Americans with the requisite cognitive skills to enter STEM careers”^5^. Simultaneously, a multitude of longitudinal and cross-sectional associational studies reported significant math-space relations, across a myriad of spatial and mathematics sub-domains, in both children and adults, even after controlling for other general reasoning factors^6,7^. This literature includes evidence that spatial and mathematical thinking both rely on shared neural substrates, namely, regions in and around the intraparietal sulcus^8^. These findings prompted a wave of intervention work exploring whether training to improve spatial skill might spontaneously transfer to gains in mathematics, with some studies showing clear evidence of transfer and others failing to demonstrate such effects.

To better understand this mixed literature and identify conditions that lead to successful transfer, our team conducted a meta-analysis_._ Our meta-analysis included 29 studies (*N* = 3765), that used rigorous pre-post designs, allowing us to make causal inferences about the effects of spatial training/instruction on mathematics performance. The results of this study were promising, with an overall effect size of 0.28 (Hedges’s *g*) for spatial training on mathematics compared to controls^9^. This finding demonstrates that spatial training improves not only spatial but also mathematics outcomes, with an effect size that compares favorably to the average effects of other randomized (0.16) and non-randomized (0.23) educational interventions^10^, and mathematics-interventions more specifically (0.06)^11^. For further contextualization, consider that the same effect sizes have been reported in the annual gains for standardized mathematics performance for U.S. children in Grades 6–10^12^. While these comparisons may over-estimate the power of spatial intervention, perhaps due to the use of predominantly white, educated, industrial, rich, democratic (or WEIRD) samples in research or the use of researcher-developed mathematics outcomes, our findings nonetheless suggest immense potential for spatial training in the classroom which could have substantial practical implications.

Our findings also provide evidence that not all spatial interventions are equivalent, perhaps explaining why not all spatial training studies have reported significant effects. Larger gains were found for training based on concrete materials (e.g., physical 3D shapes, manipulatives, blocks) compared to computer-based training. We also found that spatial training effects on mathematics increased with age, perhaps because older children are grappling with higher-level mathematics content that may benefit more from spatial visualization skills, or perhaps because older children are more proficient at using spatial representations strategically when attacking novel mathematics problems than younger children. From a classroom practice and policy perspective, this finding suggests that spatial intervention should not be constrained to the early years, and indeed spatial training in older age groups may lead to larger gains in mathematics. Interestingly, other moderators such as duration of training had no significant effect on transfer of spatial training gains to mathematics, suggesting that even short exposure to spatial intervention may enable children to reach a minimum threshold in spatial skill that facilitates improved mathematics performance. Taken together, these results provide insight into how educational policy might incorporate spatial instruction most effectively.

Despite the evidence outlined above, spatial reasoning remains underrepresented in many mathematics curricula worldwide. When spatial skill is included in the curriculum, it is often limited to identifying the names and describing properties of various 2D and 3D shapes, using spatial language such as *above, below, left*, and *right*, or learning formulae for calculating area, perimeter, and volume. Although learning objectives that refer to “*shape*” are often perceived as inherently spatial, many tasks such as naming shapes or learning shape properties, require limited, if any, spatial skill. These learning objectives predominantly call on memory and verbal recall skills, and place little emphasis on the dynamic and imaginative types of spatial thinking found to support mathematics learning. Indeed, few policy makers have explicitly prioritized spatial reasoning skills such as spatial visualization, i.e., the ability to generate and mentally manipulate objects or images, in mathematics curricula. Spatial visualization is a valuable tool for mathematical problem solving, as it can be strategically used as a “mental blackboard” to model, simulate and manipulate mathematical problems and relations. This is also true for problem solving in other STEM domains. As shown in Fig. 1, activities that target spatial visualization skills include mental rotation (mentally turning objects/shapes by various degrees of rotation), mental transformation (mentally combining or deconstructing shapes within or across planes), perspective taking (imagining scenes/objects from different points of view) and spatial scaling (mentally mapping between spaces of different scales), among others. These activities are seldom referenced in mathematics curricula. For example, none of these spatial skills are mentioned in the Common Core State Standards for Mathematics, used by many states in the U.S., where the primary focus of geometry standards is on classifying and identifying simple and complex shapes. Similarly, while the 2013 primary school (age 5–11 years) mathematics curriculum in England (UK) refers to rotation, this is in the context of right angles and half and three-quarter turns (such as on a clock face) and not the practice of *mental* rotation. The 2014 Australian primary mathematics curriculum has more spatial content within its “Measurement and Geometry” subdomain, including reference to location and transformation (e.g., map reading and giving and receiving directions) and manually cutting, folding, and turning shapes. However, even here the focus is on physical action with shape with limited attention to *mental* processing.

**Fig. 1:**
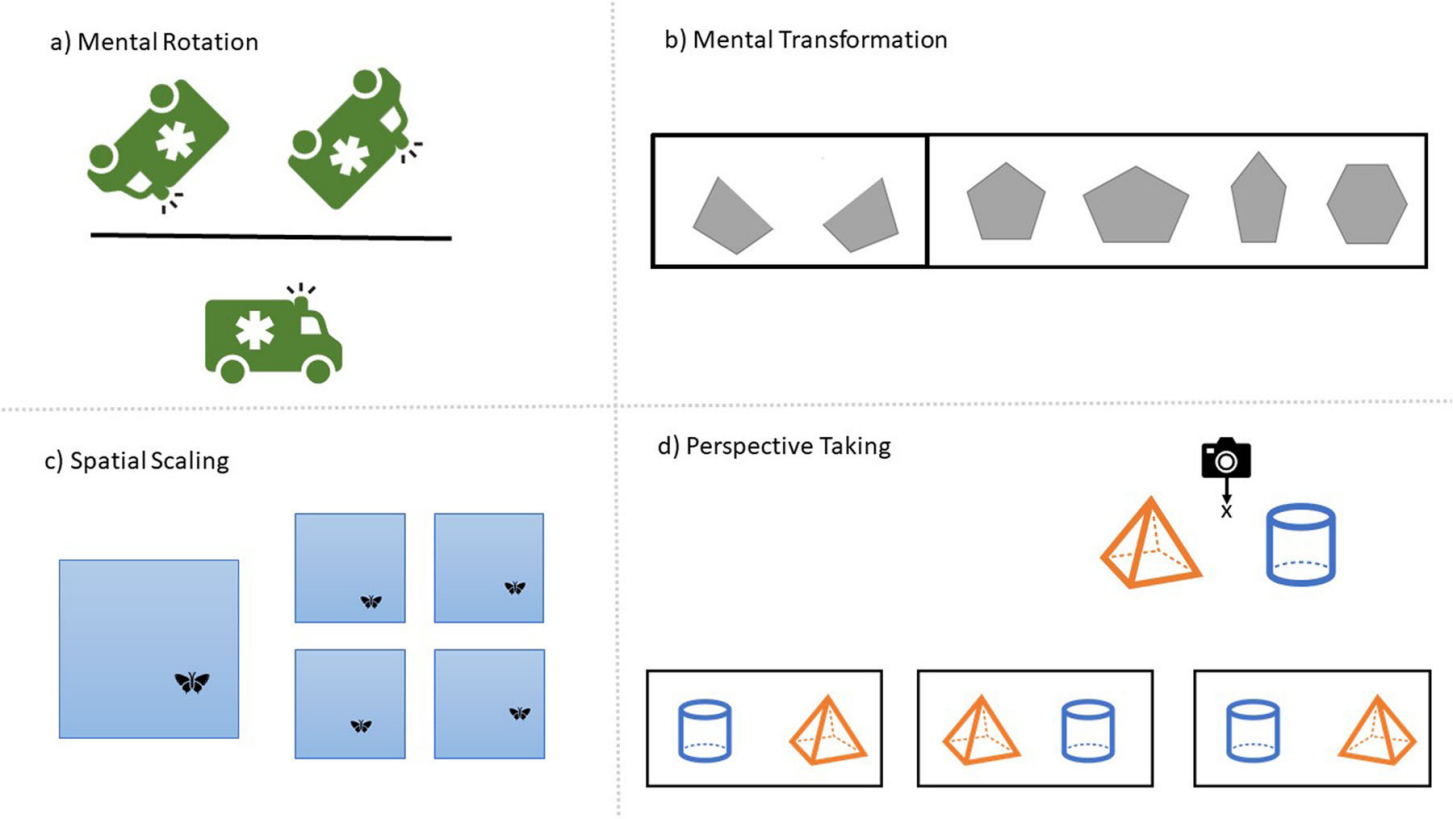
Sample spatial tasks used to assess different spatial skills. **a** Mental Rotation, participants must choose which image above the line is the same as the image below the line. **b** Mental Transformation, participants must select which shape on the right can be formed by combining the two shapes on the left. **c** Spatial Scaling, participants must select which of 4 small pictures shows the butterfly in the same position in the sky as the larger picture. **d** Perspective Taking, participants must choose which photograph from the three options at the bottom, would be taken if a photographer was standing at position x.

It is noteworthy that some countries have recently added spatial reasoning to their mathematics curricula. For example, the 2020 Ontario (Canada) Mathematics curriculum explicitly highlights spatial reasoning as a gateway to success in STEM domains and includes a “spatial sense” sub-domain for all year groups. However, other policymakers are actively reducing the attention given to spatial thinking in the classroom. For example, from September 2021, the Early Years Foundation Stage statutory framework (learning standards for children from birth to 5 years) in England, has been revised to remove early learning goals related to shape and measurement, in place of increased emphasis on number skills. As outlined above, while some *“shape”* based learning goals are currently at the mercy of predominantly ‘non-spatial’ approaches to instruction, it would be a mistake to eliminate these aspects of the curriculum completely. Instead, these aspects of the mathematics curriculum offer a logical starting place and plentiful opportunities to further ‘spatialize’ the mathematics curriculum. Rather than do away with these curriculum strands, we should be finding ways to leverage their inherently spatial properties and look to promote both spatial and mathematical thinking simultaneously (e.g., see ref. ^13^). Moreover, educational practitioners may perceive shape and space activities to be synonymous with spatial reasoning. As such, the removal of early learning goals related to shape and measurement may be interpreted as an active de-prioritization of spatial reasoning in the classroom, which reflects a wider misunderstanding of the value of spatial reasoning for mathematics achievement. Mathematics experts in England have already expressed concerns about the omission of spatial reasoning from government recommendations for the Early Years^14^. This has led to developments such as the creation of a Spatial Toolkit by the Early Years Mathematics Group^15^ to help support teachers in their use of spatial reasoning in the classroom, without specific curriculum guidance.

Students from many countries, including the UK and US, show relative weaknesses in shape and space domains on international assessments of mathematics (e.g., PISA and TIMMS) compared to other mathematics sub-domains^16^ suggesting that spatial instruction should be increased rather than eliminated. The impact of weak or non-existent spatial instruction is further pronounced when one considers preliminary evidence that the associations between spatial skill and mathematics may be particularly strong in children from lower socio-economic status (SES) backgrounds^17^ and that spatial instruction may be particularly effective for improving mathematics for pre-schoolers from lower SES families^18,19^. These findings may reflect lower starting points in spatial skill for children from lower SES families compared to their higher SES peers, which may be attributable to reduced access to spatial toys and resources, or lower quality of spatial play for children from lower SES groups. At the same time, research also demonstrates that children of lower SES backgrounds present relative strengths in spatial thinking compared to more traditional academic subjects of literacy and numeracy^20^. This evidence suggests untapped, hidden strength and potential in populations of students’ routinely underserved in current educational systems. Although further evidence is needed, these early findings point to the possibility that the removal of spatial learning objectives from early years classrooms may have a disproportionate negative influence on children from lower SES families, a subgroup of children that is already more likely to fall behind their peers. An increased focus on spatial learning and instruction may offer children from under-resourced community’s new opportunities to engage, learn, and showcase their strengths in the mathematics classroom.

Clearly, curricular changes are needed to bring spatial thinking back into the curriculum, but to fully realize its potential, changes may also be needed at the level of educational leadership and perhaps teacher training/professional development. There is considerable variability in teachers’ preparation to engage spatial reasoning. Many teachers do so effectively despite the lack of curricular guidance, particularly when teaching STEM topics^21,22^. However, this is not always the case. Research has revealed that some teachers avoid spatial activities due to weaknesses in their own spatial reasoning^20,21,23^ or anxiety they feel toward spatial tasks^21,24^. These teachers may need additional support and training to both improve their own spatial skills and master new spatially-based instructional techniques. Educational leaders can also support teachers who are already successful at implementing spatial instruction, by giving them permission to prioritize spatial thinking and helping them find efficient ways to integrate spatial thinking with their other curricular goals. Without such support, teachers may feel they cannot justify the time spent teaching spatial thinking skills while feeling pressured to focus on other components of mathematics learning, e.g., number skills.

Given the known importance of spatial skills for STEM outcomes and the increasing demand for suitably qualified STEM employees, it is remiss to continue to ignore spatial skill development in the classroom – it is time to make spatial skills an educational priority.

## Reporting summary

Further information on research design is available in the Nature Research Reporting Summary linked to this article.

## Supplementary information

Reporting Summary

## Data Availability

There is no data associated with this manuscript.
